# Characterization of transgenic rice expressing fusion protein Cry1Ab/Vip3A for insect resistance

**DOI:** 10.1038/s41598-018-34104-4

**Published:** 2018-10-25

**Authors:** Chao Xu, Jiahui Cheng, Haiyan Lin, Chaoyang Lin, Jianhua Gao, Zhicheng Shen

**Affiliations:** 1 State Key Laboratory of Rice Biology, Institute of Insect Sciences, College of Agriculture and Biotechnology, Zhejiang University, Hangzhou, China; 2 College of Life Science, Shanxi Agricultural University, Taigu, China

## Abstract

Management of resistance development of insect pests is of great importance for continued utilization of Bt crop. The high-dose/refuge and pyramid (gene stacking) strategy are commonly employed to delay the evolution of insect resistance. Due to the anticipated difficulty for deployment of mandatory refuge for transgenic crops in China, where the size of farmer is quite small, stacking of genes with different modes of action is a more feasible strategy. Here we report the development of transgenic rice expressing a fusion protein of Cry1Ab and Vip3A toxin. Analysis of trypsin proteolysis suggested that the fusion protein is equivalent to the combination of Cry1Ab and Vip3A protein. The transgenic plants expressing the fusion protein were found to be highly resistant to two major rice pests, Asiatic rice borer *Chilo suppressalis* (Lepidoptera: Crambidae) and rice leaf folder *Cnaphalocrocis medinalis* (Lepidoptera: Crambidae), while their agronomic performances showed no significant difference compared to the non-transgenic recipient rice. Therefore, the transgenic rice may be utilized for rice pest control in China.

## Introduction

*Bacillus thuringiensis* (Bt) was a ubiquitous gram-positive and sporulating bacterium that produces various insecticidal proteins. Crops have been engineered to express Bt insecticidal toxins for controlling insect pest species of Lepidoptera and Coleoptera^1–3^. The commercialization of Bt crop have delivered significant benefits to farmers during the latest 20 years^4–7^. Bt toxins have no significant risk to the environment or to human health^8^. Two sorts of insect specific toxins produced by Bt have been commercialized in agriculture, which are insecticidal crystal protein (Cry)^7,9^ and vegetative insecticidal protein (Vip)^10,11^. Most of Cry proteins are produced in parasporal crystals during sporulation^1^. To date, more than 800 Cry proteins were identified and some of them were developed commercially^12^. Vip toxins are produced during the vegetative growth stage of Bt and share no nucleotide sequence similarity to Cry proteins. Vip3A is a Vip3 toxin that is highly active to lepidopteran insects and has a totally different mode of action with Cry toxins^1,10,11^. Several Bt genes like *cry1Ab/1Ac*, *cry2A* and *cry1F*, and *vip3Aa19* are successfully engineered to generate commercial Bt transgenic events for pest control, such as Mon810, Bt11 and MIR162^4,13^.

Nevertheless, due to the long-term application, field-evolved insect resistance to Bt toxins has become a serious threat to the continued utilization of Bt crops and diminished their benefits in recent years^5,14^. A major corn pest, fall armyworm (*Spodoptera frugiperda*), was reported evolved resistance on TC1507 maize in Puerto Rico since 2006^15^. In Brazil, transgenic maize producing Bt toxin Cry1F started to be commercialized since 2008 and was found damage caused by fall armyworm in 2011^16,17^. Cotton bollworm (*Helicoverpa armigera*) and pink bollworm (*Pectinophora gossypiella*) evolved resistance on transgenic cotton expressing Cry1Ac in China during the last 10 years^18–20^. These lessons asked for improved strategy for insect resistance management. Currently, two strategies are generally considered to delay the resistance evolution^5,21^. One is the refuge strategy, which is the primary approach in insect resistance management and proven effectiveness worldwide^5,19^. However, it is a challenge in China and other developing countries due to their small farm size^22,23^. The other prevalent approach is the pyramiding (gene stacking) strategy that pyramids two or more Bt toxins killing the same insect pest. The assumption underlying this strategy is that the toxins have different modes of action and would not cause cross-resistance. Pyramided Bt crops producing two or more toxins have been rapidly replaced single-toxin Bt crops in some area, such as transgenic cotton in Australia and the United States^13,14,24^.

In China, rice (*Oryza sativa* L.) is the staple food for most people. Although transgenic rice had not been commercially planted in China, researches on Bt transgenic rice has lasted for over 20 years. The transgenic line KMD1 expressing a synthetic *cry1Ab* gene was highly resistant to eight lepidopteran rice pest species^25^. Another case was Bt shanyou-63 containing a chimeric *cry1Ab/cry1Ac* gene, which showed high protection against rice leaffolder and yellow stem borer^26^. All these lines were single-toxin Bt events. To date, there is still no report on Bt transgenic rice expressing single Vip3A toxin. As to the high probability of insect resistance, tactics for pest management must be updated. Considering the breeding pattern of rice in China, the exploration on transgenic rice lines fusing two or more toxins seems to be a more convenient method for insect resistance management^27^.

Here we reported the development of a transgenic rice line expressing a fusion protein of Cry1Ab and Vip3A. The truncated and active *cry1Ab* gene, encoding N-terminal 651 amino acid residues of Cry1Ab, was fused in reading frame to the 5′ end of the synthetic *vip3A* gene encoding 790 amino acid residues^28^. Proteolysis of the fusion protein by trypsin suggested that it would have an equivalent activity with individual Cry1Ab and Vip3A toxin in insect midgut. Bioassay results on transgenic events revealed that the selected event A1L3 had strong insecticidal activities against two major rice pests in China, Asiatic rice borer *Chilo suppressalis* (Lepidoptera: Crambidae) and rice leaf folder *Cnaphalocrocis medinalis* (Lepidoptera: Crambidae). Moreover, the insect resistance trait of A1L3 was found to be stable among plants of different generations. Thus, the transgenic line A1L3 could be a good candidate for rice pest control in China.

## Results

### Fusion protein expression and its insecticidal activity

**Figure 1:**
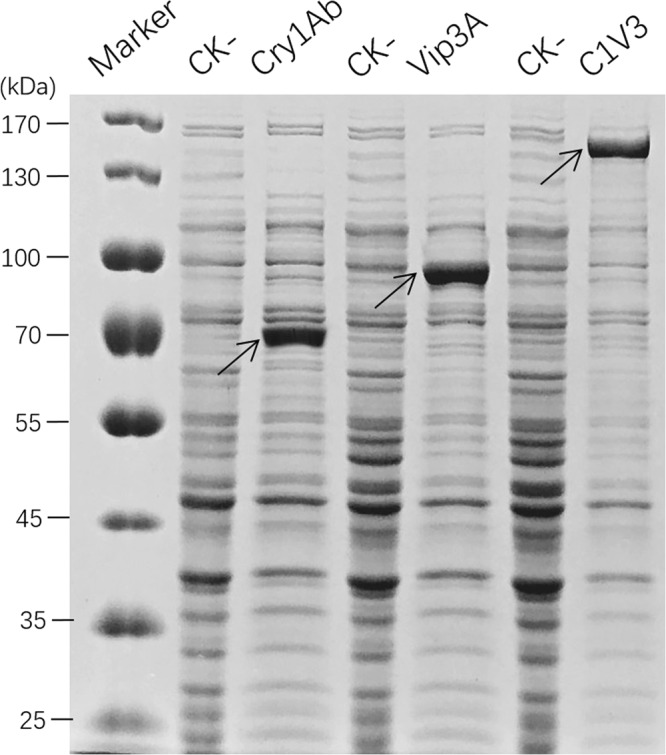
Sodium dodecyl sulfate–polyacrylamide gel electrophoresis analysis of recombinant insecticidal protein expressed by *E. coli.* Cry1Ab, Vip3A and C1V3 were all expressed by *E. coli* using pET28a vector. CK−, *E. coli* cultures without IPTG induction.

**Figure 2:**
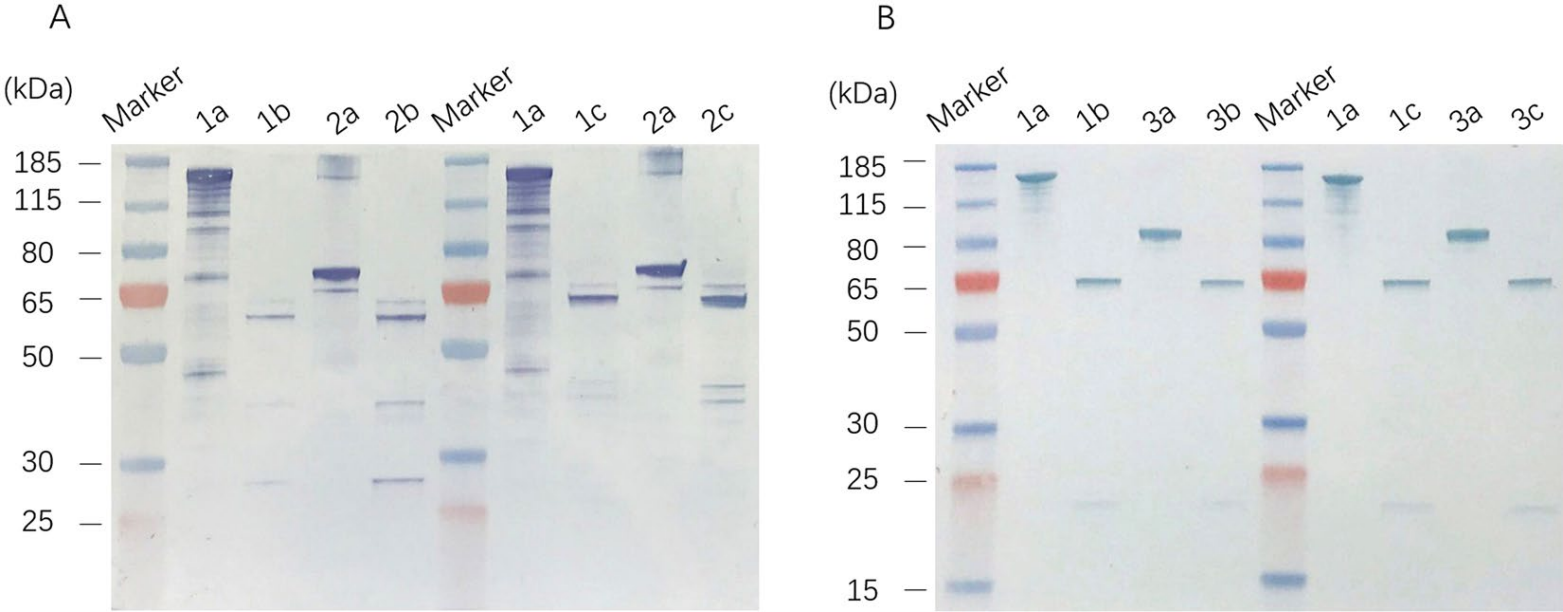
Western blot analysis of trypsin and midgut extraction digested products of Cry1Ab, Vip3A and C1V3. (**A**) polyclonal antisera against Cry1Ab was used as the first antibody. (**B**) polyclonal antisera against Vip3A was used as the first antibody. Lane 1a, C1V3 protein incubated in 37 °C without trypsin for 2h. Lane 1b, C1V3 protein incubated with trypsin in 37 °C for 2h. Lane 1c, C1V3 protein incubated with midgut juice in 37 °C for 2h. Lane 2a, Cry1Ab protein incubated in 37 °C without trypsin for 2h. Lane 2b, Cry1Ab protein incubated in 37 °C with trypsin for 2h. Lane 2c, Cry1Ab protein incubated with midgut juice in 37 °C for 2h. Lane 3a, Vip3A protein incubated in 37 °C without trypsin for 2h. Lane3b, Vip3A protein incubated with trypsin in 37 °C for 2h. Lane 3c, Vip3A protein incubated with midgut juice in 37 °C for 2h.

The truncated *cry1Ab* and the full-length *vip3A* gene were fused by a 24 base-pair nucleotide linker in reading frame to generate *C1V3* gene. This fusion gene was inserted into pET28a vector and then transformed into *E. coli* BL21(DE3) strain for protein over expression. *E. coli* expressed protein was examined by sodium dodecyl sulfate–polyacrylamide gel electrophoresis analysis (SDS-PAGE, Fig. 1). The result showed that the C1V3 protein was expressed at high level as inclusion body. The molecular weight of expressed C1V3 was about 160-kDa as expected. When digested with trypsin, active Cry1Ab and Vip3A protein generated trypsin-resistant core of about 60-kDa and 65-kDa in size, respectively^2,10^. These trypsin-resistant cores are the hallmark of active Bt toxins. A commercial trypsin and the insect midgut juice was prepared to investigate if C1V3 protein retained these trypsin-resistant cores, and then Western blot analysis was performed with Cry1Ab and Vip3A polyclonal antiserum respectively (Fig. 2). We found that C1V3 protein did generate a ~60-kDa Cry1Ab and a ~65-kDa Vip3A trypsin-resistant core, exactly same as individual Cry1Ab and Vip3A were digested by trypsin and insect midgut juice (Fig. 2). Therefore, once the fusion protein is ingested by insects, it could be processed to activated cores and works like a combination of two individual proteins of Cry1Ab and Vip3A.

### Rice transformation and screening

**Figure 3:**
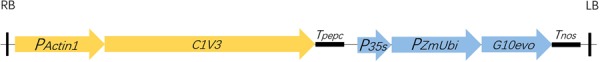
Diagram of T-DNA for expression of cry1Ab/vip3A fusion gene. The vector contains an insect resistance cassette and a glyphosate selection maker cassette. PActin1, rice actin 1 promoter. C1V3, the fusion gene of cry1Ab/vip3A. Tpepc, maize PEPc terminator. P35s, CaMV 35s promoter. PZmUbi, maize polyubiquitin-1 promoter. G10evo, a glyphosate-resistance gene. Tnos, Agrobacterium tumefaciens nos terminator. RB, right border of T-DNA. LB, left border of T-DNA.

A binary vector was constructed for rice transformation based on vector pCambia1300. Two functional cassettes were constructed into T-DNA of the vector. One is the fusion Bt gene for insect resistance and the other is the EPSPS gene for glyphosate tolerance (Fig. 3). Approximately 100 transgenic T_0_ lines were obtained by agrobacterium-mediated transformation. All of T0 lines was screened by enzyme-linked immunosorbent assay of Cry1Ab (ELISA, Data not shown). Transgenic lines with C1V3 concentration lower than 4.0 μg per gram of fresh leaf weight were discarded. Ten transgenic lines were selected for further analysis with expression level from 4.0 to 28 μg per gram of fresh leaf weight. The T-DNA border sequence of most of these lines were then determined. Based on the insertion site and insect resistance bioassay, a line named A1L3 was selected for further characterization and evaluation.

### Protein immunoblot analysis and quantification in generations of transgenic rice

**Figure 4:**
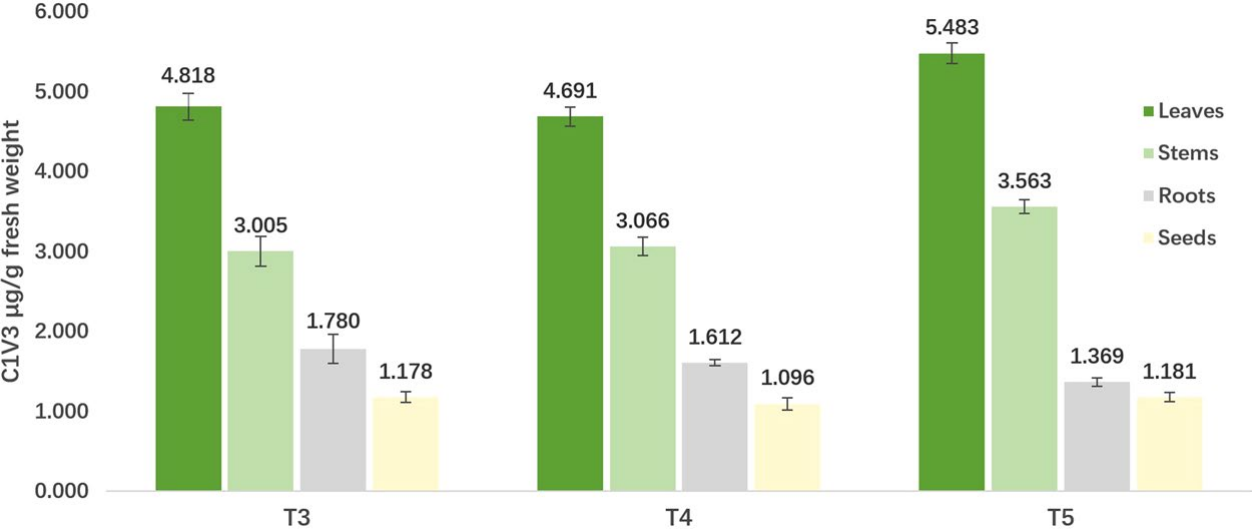
Expression levels of the fusion Bt protein in different tissues of the T3, T4 and T5 progenies of transgenic A1L3 rice at tilling stage. ELISA kit for Cry1Ab/1Ac were used to quantify the expression of insecticidal fusion protein in plants.

The expression level of T3, T4 and T5 progenies of A1L3 at tilling stage were determined by ELISA method (Fig. 4). Different tissues of each plant were sampled and measured. The C1V3 protein was expressed at 4.691~5.483 μg/g fresh leaf weight in leaves, 3.005~3.563 μg/g in stems, 1.369~1.780 μg/g in roots and 1.096~1.181 μg/g in seeds. We also found that the C1V3 protein expressed stably in different generation of transgenic rice.

**Figure 5:**
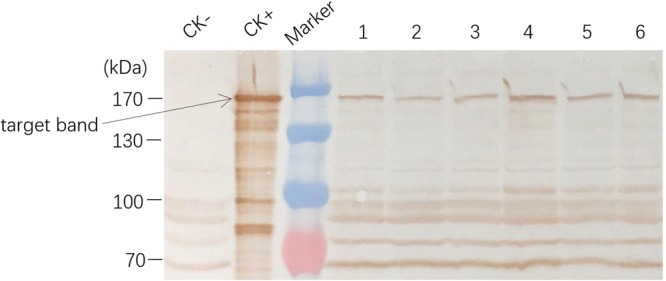
Western blot analysis of the Bt fusion protein in transgenic rice. Polyclonal antisera against Vip3A was used as the first antibody. CK−, negative control of non-transgenic rice. CK+, *E. coli* expressed fusion protein C1V3 used as the positive control. Lane 1–6, six samples extracted from rice leaves. The arrow mark indicated the target insecticidal fusion protein C1V3.

We also analyzed C1V3 protein by Western blot analysis(Fig. 5). Six A1L3 samples from different plants of the T5 generation were selected for detection of the fusion protein. We detected the fusion protein in plant leaves and found that it has almost identical size of the *E. coli* expressed C1V3 protein, about ~162-kDa (Supplementary Fig. S1).

### T-DNA integration in transgenic rice genome

**Figure 6:**
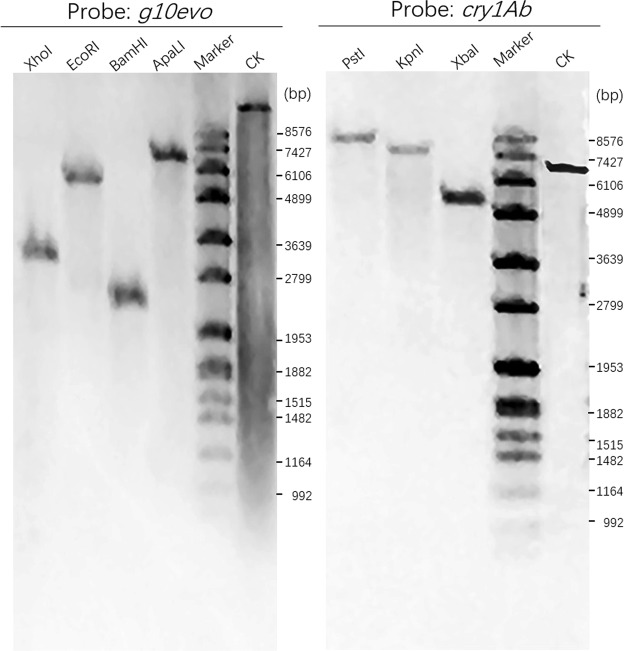
Southern blot analysis of transgenic line A1L3. Digoxin labeled dsDNA of g10evo and cry1Ab were used as probes to detect T-DNA. For each probe, the genomic DNA was digested by three or four different restriction enzymes respectively. CK, plasmid containing g10evo or cry1Ab gene served as positive control.

The insertion site and copy number of T-DNA were analyzed by hiTail-PCR and Southern blot, respectively. Sequencing results suggested that an intact T-DNA was inserted at the Chromosome 3. The Southern blot analysis showed only one band for each enzyme-digested sample, indicating that there was only one T-DNA copy in the rice genome (Fig. 6).

### Evaluation of insect resistance of A1L3 plants

Two major species of rice lepidopteran pests, Asiatic rice borer (*C. suppressalis*) and rice leaf folder (*C. medinalis*), were selected for insect bioassay. A1L3 showed high resistance to both insect species at different developmental stages. For *C. suppressalis* neonates transferred on rice leaves at seedling, tilling, and filling stage, mortality was measured at 84.4%, 89.4% and 41.3% respectively after fed for 48 h, and the mortality for all 3 tissues reached to 100% after 96 h infestation (Table 1). For *C. medinalis* neonates transferred on rice stems at seedling, tilling, and filling stage, mortality was measured at 59.8%, 66.9%, and 35.8% respectively after feeding for 48 h., and the mortality for all 3 tissues reached to 81.3%, 100% and 87.2% after 96 h, and all of them reached 100% after 144 h (Table 1). The tested insects were all observed dead after then. The mortality of both neonate larvae fedding on the non-transgenic control was less than 5% throughout the bioassay. The leaves and stems of non-transgenic control infested by *C. suppressalis* or *C. medinalis* were significantly damaged.

**Table 1:** Mortality of *C. suppressalis* and *C. medinalis* infested on A1L3 leaves and stems respectively.

	Rice line	*C. suppressalis*			*C. medinalis*		
		Mortality (%)			Mortality (%)		
		48 h	96 h	144 h	48 h	96 h	144 h
Seedling stage	A1L3	84.4	100	100	59.8	81.3	100
	Xiushui-134	2.91	4.85	4.85	1.86	3.74	4.67
Tilling stage	A1L3	89.4	100	100	66.9	100	100
	Xiushui-134	1.33	3.99	3.99	1.89	3.77	4.72
Filling stage	A1L3	41.3	100	100	35.8	87.2	100
	Xiushui-134	1.79	2.68	3.57	0.96	2.88	4.81

### Agronomic performance of transgenic line A1L3

A1L3 line was planted in the experimental plots for two consecutive years (2016~2017) to evaluate the agronomic traits, including plant height, panicles per plant, grains per panicle, weight per 100-grain and seed-set rate. Compared to the non-transgenic line Xiushui-134, A1L3 demonstrated no significant difference (*P* > 0.05) from the control plants (Table 2).

**Table 2:** Comparison of agronomic traits between transgenic line A1L3 and non-transgenic cultivar Xiushui-134.

Rice genotype	A1L3	Xiushui-134
Plant height (cm)	82.55 ± 1.15	81.10 ± 0.85
Panicles per plant	10.64 ± 0.28	11.37 ± 0.42
Grains per panicle	135.16 ± 2.21	135.08 ± 2.46
Weight per 100-grain (g)	2.21 ± 0.13	2.17 ± 0.12
Seed-set rate (%)	88.16 ± 1.52	85.07 ± 1.29

## Discussion

Pyramiding strategy seems to be a feasible plan for rice pest resistance management in area with very small farm size. In this study, we developed a fusion strategy to express two Bt insecticidal toxins with different modes of action in transgenic rice simultaneously. A fusion gene of *cry1Ab/vip3A* with linker was utilized. Our study suggested that the fusion protein can be reconstituted into two individual toxins upon trypsin digestion. Western blot analysis revealed that C1V3 could be digested into a ~60-kDa Cry1Ab product and a ~65-kDa Vip3A product respectively, which were identical to that of the individual protein of Cry1Ab and Vip3A. The digested products by commercial trypsin and Asiatic rice borer midgut extraction indicated that C1V3 protein could successfully be activated *in vivo*. These trypsin-generated products were the hallmark of activated Bt toxins. Therefore, we suggested that C1V3 protein could be successfully activated and work equivalently to individual Cry1Ab and Vip3A. Expressing fusion protein has some advantages over expressing individual proteins. The fusion method equalizes the expression level of the two proteins, and also is more convenient in trait integration into different crop elite lines as no stacking is required.

The transgenic rice event A1L3 was selected for possible commercial development after a comprehensive evaluation of about a hundred events on insertion sites, expression levels, insect bioassay and agronomic traits. Nearly 40 events of the T0 transgenic events were discarded due to the low expression level of C1V3 (less than 4.0 μg per gram of fresh leaf weight). The C1V3 expression level of the left events was determined at the range of 4.0~28.0 μg/g-fwt. Integration of T-DNA in rice genome and bioassay on insects would be considered for events selection. Only four events were kept for further evaluation because their T-DNA didn’t integrate into any annotated loci of rice genome and the T-DNA copy number was single. A1L3 was not the highest expressers but it already demonstrated high resistance to the two major rice pests, *C. suppressalis* and *C. medinalis*. We believe that the expression level in A1L3 is high enough to control the target pests effectively. The amount of the insecticidal protein C1V3 in A1L3 was almost triple of the chimeric Cry1Ab/Cry1Ac protein expressed in previously reported transgenic events Bt Huahui-1, in which the Bt protein was determined at 1.88~2.36 μg/g fresh leaf weight^26^. We did not observe any negative effects on agronomic performances or on disease resistance in A1L3 for two consecutive years planting in fields. However, we observed in two seasons that the events with significant higher expression of C1V3 appeared to have more disease spots on leaves. We will continue our study to check if there is any relationship between the expression levels of C1V3 and the disease spots in their leaves.

## Methods

### Fusion gene assembling

The coding sequences of *cry1Ab* and *vip3A* gene were both optimized according to the codon bias of monocots and synthesized by Sangon Biotech (Shanghai, China). A BamHI and SmaI restriction site were introduced into start and end codon of *cry1Ab* respectively, as SmaI and SacI into *vip3A* while synthesizing. Meanwhile, a 24 base pair nucleotide linker was introduced to conjugate *cry1Ab* and *vip3A* gene. The linker encodes a peptide with 8 amino acids (GGAGGAGG). A pair of reverse complementary oligonucleotides linker-F (5′-GGTGGAGCAGGTGGAGCAGGTGGA-3′) and linker-R (5′-TCCACCTGCTCCACCTGCTCCACC-3′) were used to generate *linker* sequence with 72 °C extension processing for 15 mins in thermocycler. Linker was assembled to the blunt ends of *cry1Ab* (BamHI-SmaI) and *vip3A* (SmaI-SacI) fragment.

### Expression of fusion protein in *E. coli*

The fusion gene *C1V3* fragment digested by BamHI and SacI was constructed into expression vector pET28a (Novagen, San Diego, USA). The expression vector was transformed into *E. coli* BL21 (DE3) strain (Stratagene, Santa Clara, USA). Single *E. coli* colony harboring the *pET28a-C1V3* vector was picked and incubated in Luria-Bertani (LB) liquid medium at 37 °C with shaking at 220 rpm. When the OD_600_ (optical density at 600 nm) of the cell culture reached 0.6, isopropyl β-D-1-thiogalactopyranoside (IPTG) should be added into the LB medium to 0.5 mM final concentration for inducing expression for 8 h at 16 °C with shaking at 220 rpm. Cells were collected by centrifugation at 3000 × g for 10 min at 4 °C, and then resuspended in phosphate buffered saline buffer (PBS, 137 mM NaCl, 2.7 mM KCl, 10 mM Na_2_HPO_4_, and 2 mM KH_2_PO_4_, pH 7.4) and sonicated for 30 min on ice. Subsequently, the total sonicated cell lysate was centrifuged at 10000 × g for 15 min. The supernatant and the inclusion body should be stored separately in equal volume of PBS buffer, as usual as 10 ml. Samples were examined by sodium dodecyl sulfate–polyacrylamide gel electrophoresis (SDS-PAGE) analysis with Coomassie brilliant blue (CBB) dying and densitometrically quantified in gel using a GS-800 imaging system and Quantity One image analysis software from Bio-Rad Laboratories (Hercules, USA). The *E. coli* expressed protein was digested by trypsin (Trypsin from bovine pancreas, Sigma-Aldrich, Catalog number T8802) and insect midgut juice. Protein suspension was incubated with trypsin in a final concentration of 0.5 μg/μl at 37 °C for 0.5 min, 5 min, 15 min, 30 min, 1 h and 6 h to estimate the digestion process. In our assay, 1 h was enough to convert all of the full-length ~160-kDa protein into presumed ~60-kDa active cores of Cry1Ab and Vip3A. The insect midgut juice was extracted from five-star larvae of Asiatic rice borer. Proteins were incubated with the midgut juice extraction at a ratio of 3:1 (e.g. 75 μl protein suspension with 25 μl midgut juice, 100 μl in total) at 37 °C for 2 h. After incubation, digestion reaction was immediately terminated by protease inhibitor cocktail (MedChemExpress, Shanghai). Western blot analysis was utilized to identify the digestion products using specific polyclonal antisera. All of the polyclonal antisera were personalized and manufactured by GenScript Biotech Corp. (Service No. SC1030, Nanjing, China).

### Construction of binary vectors for transformation

The vector for rice transformation was based on pCambia1300 (Cambia, Canberra, Australia). A herbicide-resistance gene *g10evo* encoding a glyphosate-tolerant 5-enolpyruvylshikimate-3-phosphate synthase (EPSPS) substituted the original *hptII* as a selection marker gene for plant tissue culture. *Zea mays* polyubiquitin-1 promoter *PZmUbi-1* (Genbank No: S94464) and the cauliflower mosaic virus promoter *P35S* were fused as the synthetic promoter in the glyphosate-tolerance expression cassette. The C1V3 cassette containing *Oryza sativa* actin gene promoter *PActin1* (Genbank No: NC_008398) and maize phosphoenolpyruvate carboxylase terminator *Tpepc* (Genbank No: X15239) was inserted into the multiple cloning site. *PZmUbi-1* was cloned from corn genome using a pair of primer pUbi-F (5′-CTAAGCTTGCATGCCTACAGTGCAGCGTGA-3′, *Hind*III restriction site underlined) and pUbi-R (5′-ATGGATCCTCTAGAGTCGACCTGCAGAAGTAAC-3′, *BamH*I restriction site underlined). *PActin1* was cloned from rice genome using a pair of primer pAct-F (5′-CTAAGCTTAGGTCATTCATATGCTTGAGAAGAGTC-3′, *Hind*III restriction site underlined) and pAct-R (5′-ATGGATCCTCGGCGTCAGCCATCTTCTAC-3′, *BamH*I restriction site underlined).

### Rice transformation

An elite local japonica rice cultivar Xiushui -134, kindly provided by Jiaxing Academy of Agricultural Science in Zhejiang Province, was used as explant for Agrobacterium-mediated transformation. The binary vector carrying the insect-resistance gene and the selection marker cassettes were transformed into *A. tumefaciens* strain LBA4404 through electroporation. Plant tissue culture method was totally folllwed as Hiei Y. *et al*.^29^, except that 2 mM glyphosate (Sigma-Aldrich, St. Louis, USA) was used for selection.

### Protein quantification in transgenic events

Leaves in the tiller stage of transgenic rice events were picked to measure the concentration of C1V3 protein by enzyme-linked immunosorbent assay (ELISA). A commercial ELISA Kit for Cry1Ab/Cry1Ac (Envirologix QualiPlate™, CA, USA) product was utilized to measure the soluble target protein concentration in plant tissues. All procedures followed according to correspondent users’ manual guide.

### Western blot analysis

To detect the expression of C1V3 protein in transgenic lines, Western blot analysis was utilized. Protein was extracted from fresh leaves in the tiller stage and dissolved in PBS buffer (pH 7.4) with protease inhibitor cocktail (MedChemExpress, Shanghai, China). Custom-made rabbit antiserum against Cry1Ab or Vip3A was used as the primary antibody, and the horseradish peroxidase-conjugated goat anti-rabbit IgG was used as the secondary antibody. TMB stabilized substrate solution for Horseradish Peroxidase (Promega, catalog number W4121) or 3,3′-diaminobenzidine tetrahydrochloride (DAB, Sigma-Aldrich #D5905,) was used for detection. PageRuler prestained Protein Ladder (Thermo Scientific, product# 26616 & 26619) were used as marker.

### Southern blot analysis

To detect T-DNA insertion in transgenic rice genome, Southern blot was utilized. Firstly, we amplified the flanking sequence to identity the border sequence of T-DNA using hiTAIL-PCR method^30^. Depending on the flanking sequence, the location of T-DNA on rice genome was found. Thus we predicted the length of DNA containing probe sequence (*cry1Ab* gene and *g10evo* gene respectively) digested by appropriate restriction enzymes. For *g10evo* nucleotide probe, rice genome was digested by four restrict enzymes (XhoI, EcoRI, BamHI and ApaLI) respectively. For *cry1Ab* nucleotide probe, rice genome was digested by three restrict enzymes (XbaI, PstI and KpnI) respectively. Primers used to amplify the fragments of probes was displayed in Supplementary Table S1. Protocol was demonstrated as Users’ manual (Version 13) of DIG High Prime DNA Labeling and Detection Starter Kit II (Roche).

### Insect bioassays

Insecticidal activities were evaluated for transgenic rice and carried out as described by Ye *et al*.^31^. Bioassay would performed 30 replicates for both transgenic samples and non-transgenic control respectively. Insect eggs were provided by Genralpest Biotech (Beijing, China), which colony was reared on artificial diet for 10–20 generations. For bioassay of transgenic rice against Asiatic rice borer *C. suppressalis* (Lepidoptera: Crambidae), neonate larvae (1–2 hr old) were fed on fresh leaves at stem elongation stage, while non-transgenic rice Xiushui-134 at the same growth period was used as negative control. 75 mm-diam petri dishes with a filter paper on the bottom were prepared for cultivation. For each replicate, 10 larvae were introduced into each petri dish with a piece of 50–60 mm leaf, and 200 μl ddH_2_O was dropped on the filter paper to maintain humidity. Subsequently, parafilm membrane was used to seal the petri dishes to prevent larvae from escaping. All petri dishes were stored in a hermetic box without light at 28 °C. For each replicate of bioassay against rice leaf folder *C. medinalis* (Lepidoptera: Crambidae), 15 neonate larvae were placed on the living plant leaves of transgenic rice and the non-transgenic control respectively. All samples were placed in the cage in greenhouse.

### Evaluating the agronomic performance

Transgenic rice lines were planted in paddy field at the Transgenic Experimental Plots of Zhejiang University (Changxing, Zhejiang, China) to evaluate the agronomic performance. Non-transgenic control line Xiushui-134 was planted in paddy field adjacent to the transgenic lines. Six blocks of 6 m^2^ (2 m × 3 m) were chosen randomly for the evaluation. Each block contained about 100 plants and each plant had about 10–15 tillers. Five agronomic traits were measured, including plant height, panicles per plant, grains per panicle, weight per 100-grain and seed-set rate. Transgenic plants agronomic traits were compared with Xiushui-134 using student’s *t*-test. Values were given as means (±SD).

## Electronic supplementary material

Supplementary information

## Data Availability

All data generated or analysed during this study are included in this published article.
