# Supplementary material for: Characterization of transgenic rice expressing fusion protein Cry1Ab/Vip3A for insect resistance
Source: Sci Rep. 2018 Oct 25;8:15788. doi: 10.1038/s41598-018-34104-4 (PMC6202352; doi:10.1038/s41598-018-34104-4)
Supplement: Supplementary file 1 — Supplementary information [file 41598_2018_34104_MOESM1_ESM.pdf]

Characterization of transgenic rice expressing fusion protein Cry1Ab/Vip3A for insect resistance

Authors: Chao Xu[1], Jiahui Cheng[1], Haiyan Lin[1], Chaoyang Lin[1], Jianhua Gao[2], Zhicheng Shen[1*]

1 State Key Laboratory of Rice Biology, Institute of Insect Sciences, College of Agriculture and Biotechnology, Zhejiang University, Hangzhou, China

2 College of Life Science, Shanxi Agricultural University, Taigu, China

* Correspondence and requests for materials should be addressed to Z.S. (email: zcshen@zju.edu.cn)

Supplementary Figure S1

Western blot analysis of the Bt fusion protein in transgenic rice. Polyclonal antisera against Vip3A was used as the first antibody. CK-, negative control of non-transgenic rice. CK+, *E. coli* expressed fusion protein C1V3 used as the positive control. Lane 1-6, six samples extracted from rice leaves. The arrow mark indicated the target insecticidal fusion protein C1V3.

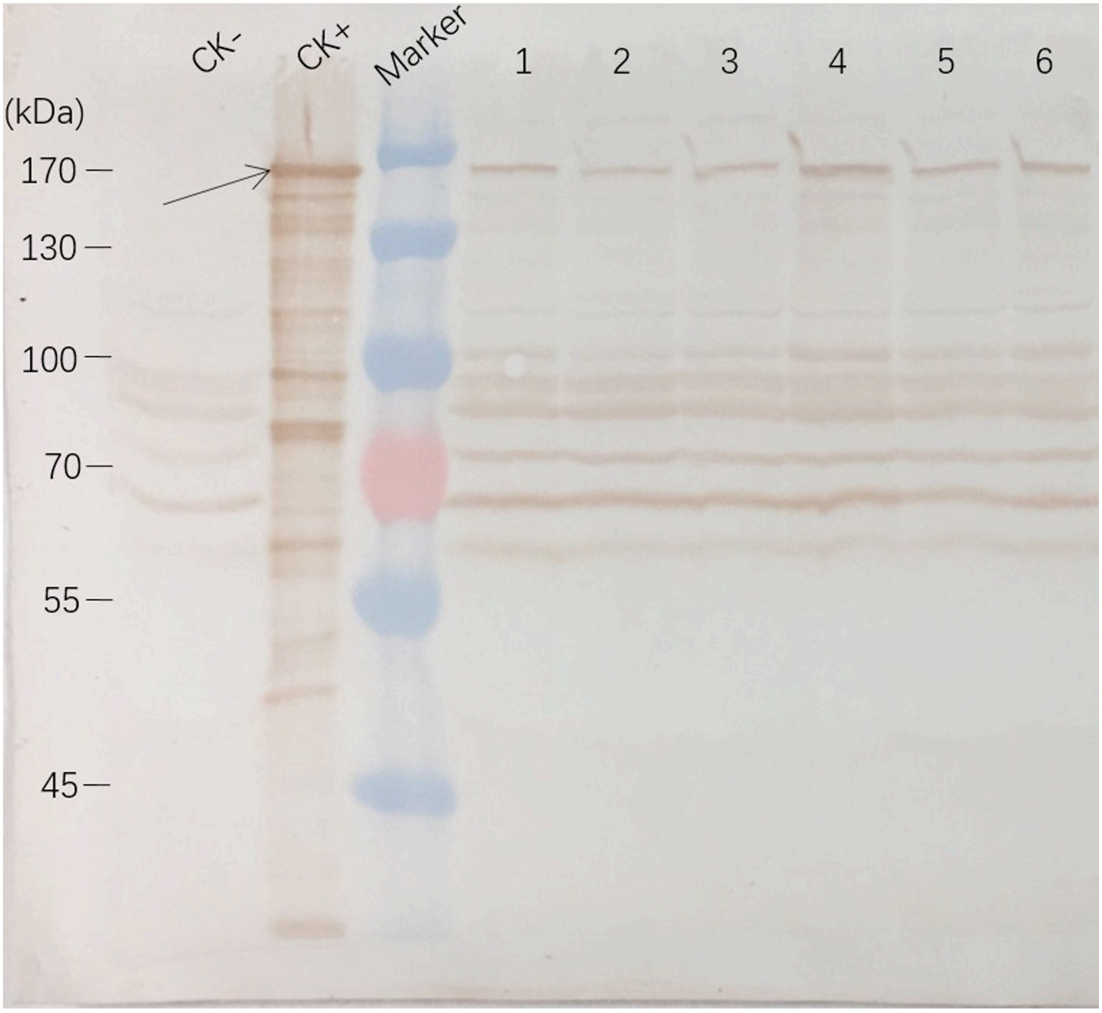

Supplementary Figure S2

The original exposures of Southern blot on A1L3. A, digoxin labeled dsDNA of g10evo was used as probes to detect

T-DNA. B1 & B2, digoxin labeled dsDNA of cry1Ab was used as probes to detect T-DNA. While CK was over –exposed for long-exposure (B1), a short-exposure (B2) was performed as well to detect CK sample.

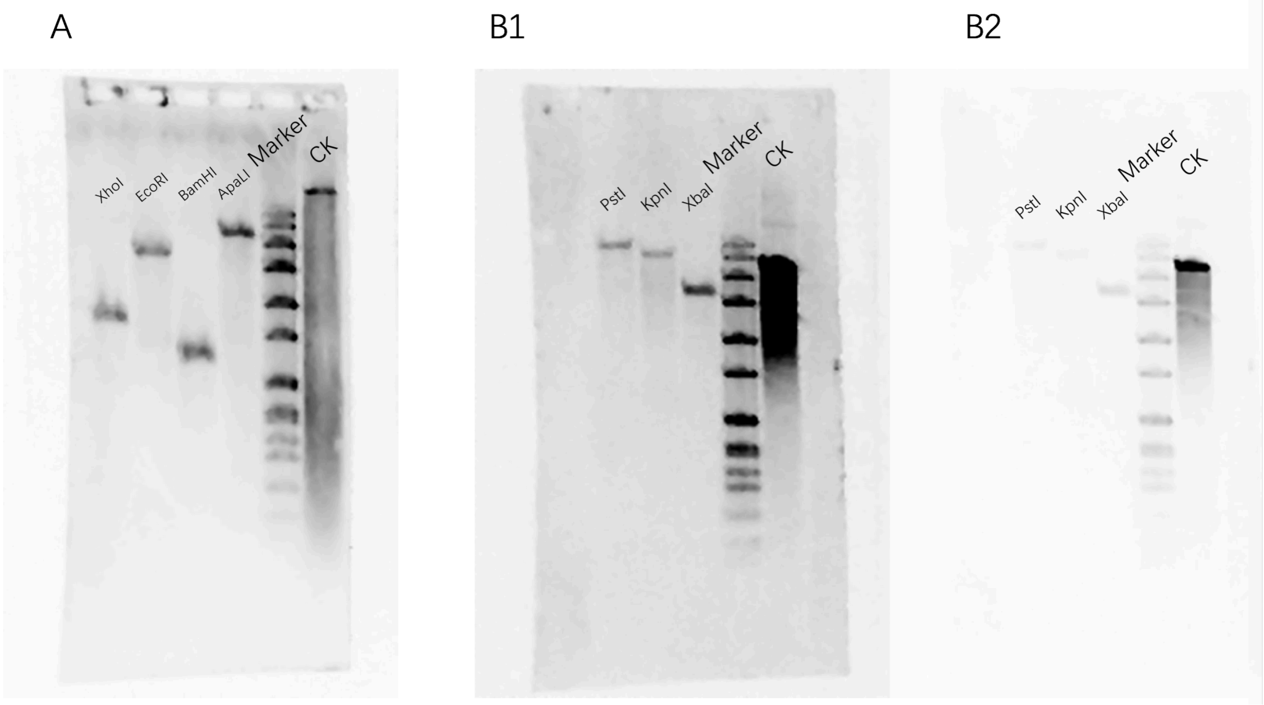

Supplementary Figure S3

Wstern blot analysis of trypsin-digested product of C1V3 protein in time gradient. C1V3, *E.coli* expressed C1V3 protein incubated in 37C° for 2h without trypsin. C1V3 samples were incubated with trypsin in 37C° in a time gradient of 0.5min, 5min, 15min, 30min, 1h and 6h. A, polyclonal antisera against Cry1Ab was used as the first antibody. B, polyclonal antisera against Vip3A was used as the first antibody. C1V3 protein could be completely digested in 1h by trypsin.

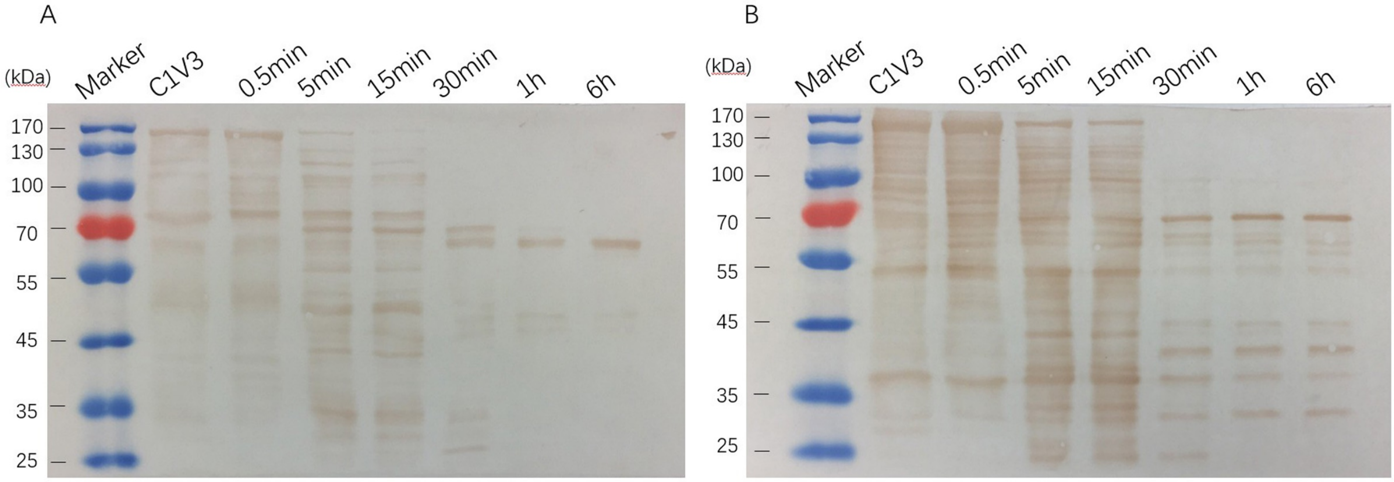

Supplementary Table S1

Primers designed to amplify the fragments for probes of Southern blot.

	Sequences
G10-probe-F	5'- CACCTTCGACGTGATCGTGCATCCA -3'
G10-probe-R	5'- CGAGGTGAGCGAAGAACTGAGGGTAGGA -3'
1Ab-probe-F	5'- GACAACAACCCCAACATCAACGAGTG -3'
1Ab-probe-R	5'- GGTAGTCGGTCACGTCGGTCTT -3'
